# The homeodomain-interacting protein kinase Hipk promotes apoptosis by stabilizing the active form of Dronc

**DOI:** 10.1038/s41420-025-02916-9

**Published:** 2025-12-16

**Authors:** Juan Manuel García-Arias, Rafael Alejandro Juárez-Uribe, Luis Alberto Baena-López, Ginés Morata, Ernesto Sánchez-Herrero

**Affiliations:** 1 Centro de Biología Molecular Severo Ochoa (CBM), CSIC-UAM, Nicolás Cabrera 1, Universidad Autónoma de Madrid, Cantoblanco, 28049 Madrid, Spain; 2 Present Address: Department of Pathology and Laboratory Medicine, University of Rochester Medical Center, Rochester, NY USA

**Keywords:** Drosophila, Apoptosis

## Abstract

Members of the evolutionarily conserved homeodomain-interacting protein kinase (Hipk) family play a critical role in regulating essential signalling pathways involved in growth, differentiation, and apoptosis. While vertebrates have multiple *hipk* genes, *Drosophila* contains a single *hipk* ortholog, what facilitates functional analysis. We find that *hipk* is necessary for the stabilization of the initiator caspase Dronc, thus enhancing the two Dronc activities in apoptotic scenarios: the induction of the caspase cascade, and the reinforcement of JNK signalling pathway. Conversely, our data suggest that Dronc also raises the expression levels of Hipk, thereby reinforcing the apoptotic response. These findings significantly enhance our understanding of caspase regulation and position Hipk as a promising target for modulating caspase activity in a variety of biological contexts.

## Introduction

Apoptosis, one of the most prevalent forms of programmed cell death, is a conserved phenomenon by which cells are eliminated through an evolutionary conserved group of cysteine proteases, termed caspases, that dismantle the protein substrates and cause cell death [1]. Apoptosis can take place during normal development, like in the sculpting of Drosophila embryonic cephalic structures [2] or the elimination of the interdigital membranes in vertebrates [3], or be triggered by stress or tissue damage [4].

Because of the simplicity of its genetic system and its sophisticated genetic technology, *Drosophila* is a useful model to analyse the regulation of apoptosis. Within *Drosophila*, the wing imaginal disc is especially convenient for this analysis, since little developmentally programmed apoptosis exists, but shows a robust apoptosis induction in response to stressors like ionizing radiation (IR), heat shock, and others [5, 6]. Moreover, apoptosis can experimentally be manipulated by driving the expression of members of the apoptotic cascade, including the pro-apoptotic genes [7–11].

As in mammals, the apoptotic pathway in *Drosophila* engages pro-apoptotic genes, initiator and effector caspases and natural inhibitors of apoptosis such as Diap1 [1]. An important feature of the *Drosophila* apoptotic pathway is that it includes a feedback amplification loop (Supplementary Fig. 1), necessary for the full apoptotic response [12]. This loop involves the Jun N-Terminal Kinase (JNK) pathway, a versatile signalling pathway implicated in many biological processes [13] including apoptosis in response to stress [14, 15]. Upon irradiation, there is an initial apoptotic stage, triggered by the DNA damage response pathway, which induces the function of the initiator caspase Dronc and the effector caspases Drice and Dcp1 [4]. A second phase, consolidating the apoptotic response, appears to rely on a Dronc-dependent stimulation of the JNK pathway [12, 16–18]. Despite intensive research, the molecular crosstalk between major signalling pathways, such as the JNK pathway, and the core apoptotic machinery, remains poorly understood.

A group of factors involved in the regulation of JNK signalling are members of the conserved homeodomain-interacting protein kinase family, encoded by the *hipk* genes [19]. While vertebrates possess four *hipk* members (*hipk1-4*), *Drosophila* only contains one, what facilitates the experimental analysis of Hipk function. The *Drosophila hipk* gene shows the highest homology with the vertebrate *hipk2* [20], which encodes a protein known to interact with many transcription factors and to regulate numerous biological processes, including transcriptional regulation, cell proliferation and apoptosis [21]. The *hipk* gene of *Drosophila* also regulates major pathways like Notch [22], Wg [23], Hippo [24], JAK/STAT [25], and JNK [26]. Nevertheless, the molecular bases of these interactions are largely unknown, particularly in the context of apoptosis and JNK signalling regulation.

In this work we present evidence that the apoptotic activities of Dronc and the JNK signalling amplification are critically influenced by *hipk*. Specifically, our results indicate that to a large extent these effects stem from the mutual ability of Hipk and Dronc to regulate each other’s activities in vivo.

## Results

### Developmentally programmed apoptosis requires *hipk* function

Previous data indicated that *hipk* is required for the implementation of apoptosis in developmentally regulated scenarios in *Drosophila*, such as the removal of embryonic neurons or of epithelial wing cells after adult hatching from the puparium [27]. To further characterize the role of *hipk* during apoptosis we have analysed the consequences of compromising *hipk* function in two developmental contexts showing intrinsic apoptosis: the fusion of the adult abdominal hemi-segments, and the rotation of the male genitalia.

**Fig. 1:**
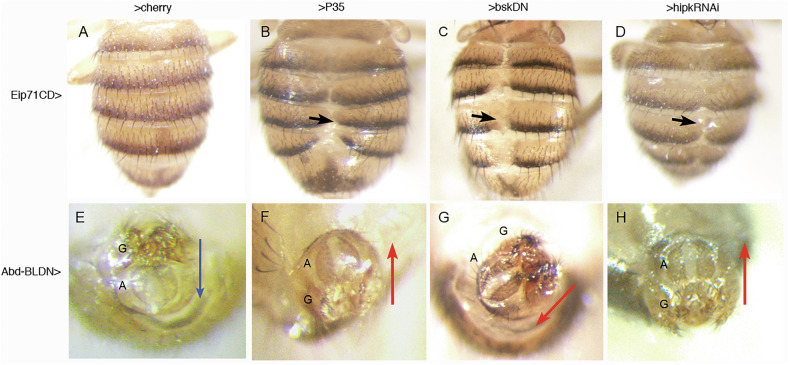
The function of *hipk* is required for developmentally programmed apoptosis in the abdomen and terminalia. **A** Control *Eip71CD*-Gal4 UAS-*cherry* female dorsal abdomen showing continuous epithelium between left and right sides of each segment. **B** When the caspase inhibitor *p35* is expressed in the LECs (*Eip71CD-Gal4 UAS-p35* flies), some left and right dorsal tergites do not meet properly at the midline (arrow). **C** Suppression of JNK activity in the larval epidermal cells (*Eip71CD-Gal4 UAS-bsk*^*DN*^ flies) causes a similar phenotype. **D** When *hipk* expression is reduced in LECs (*Eip71CD-Gal4 UAS-hipk*^*RNAi*^ flies) the phenotype is also similar, though weaker. 8–10 females were studied for each genotype, all showing a uniform phenotype. **E** Male genitalia and analia of a control *Abd-B*^*LDN*^ *UAS-cherry* fly, showing the wildtype arrangement, genitalia (G) in the upper location and analia (A) in the lower one, as indicated by the arrow from genitalia to analia. **F**, **G** in *Abd-B*^*LDN*^ *UAS-p35* (**F**) o *Abd-B*^*LDN*^ *UAS-bsk*^*DN*^ (**G**) males the normal arrangement of the analia and genitalia is reversed or abnormal, the analia now being in an anterior or lateral location with respect to the genitalia, arrows. **H** In an *Abd-B*^*LDN*^ *UAS-hipk*^*RNAI*^ male a similar abnormal phenotype is observed. 7–11 males were observed for each genotype, all with similar phenotypes. All the crosses were made at 25 °C and the larvae transferred to 31 °C to complete development.

During pupal development, polytene Larval Epidermal Cells (LECs) undergo cell death and are extruded from the epithelium. The elimination of LECs is tightly coupled with the proliferation of histoblasts that form the adult abdominal cuticle [28, 29]. The dorsal histoblasts from the left and right sides meet at the midline to form a continuous epithelium in each abdominal segment (Fig. 1A). This process depends on apoptosis-mediated elimination of the LECs, as blocking apoptosis execution by overexpressing *p35* [30] under control of the LEC-specific *Eip71CD-Gal4* driver [31] results in aberrant abdominal fusion at the midline [32]; Fig. 1B). Since JNK is an apoptosis inducer [14, 15, 18], we also examined whether this pathway was necessary for this fusion. As shown in Fig. 1C, the expression in LECS of a dominant negative form of *basket* (a key transducer of the JNK pathway), compromises the fusion between abdominal hemi-segments. Interestingly, reducing *hipk* expression in these same cells caused a weaker but comparable fusion defects to those observed after blocking apoptosis or the activity of the JNK pathway (Fig. 1D).

Next, we examined the requirement of *hipk* function for the 360° rotation of the genital plate during early pupa [33, 34]. This process locates the genitalia in the upper and the analia in the lower position of the terminalia (Fig. 1E). This arrangement requires apoptosis in the eighth abdominal segment (A8) of the male genital disc, since there is impaired or absent rotation when apoptosis is suppressed [35–38]. The overexpression of the cell death inhibitor *p35* or of a dominant negative form of *basket* with an A8-specific Gal4 line (*Abd-B*^*LDN*^) caused analia and genitalia rotation defects (Fig. 1F, G). We find that compromising *hipk* function by using the *hipkRNAi* construct with the same driver also resulted in similar rotation defects (Fig. 1H).

Taken together, these results establish that both the fusion of abdominal histoblasts nests and the rotation of the male genital plate require *hipk* function, likely by contributing to reach the apoptosis levels necessary to complete those processes.

### Experimentally induced apoptosis triggered by pro-apoptotic genes requires *hipk* activity

**Fig. 2:**
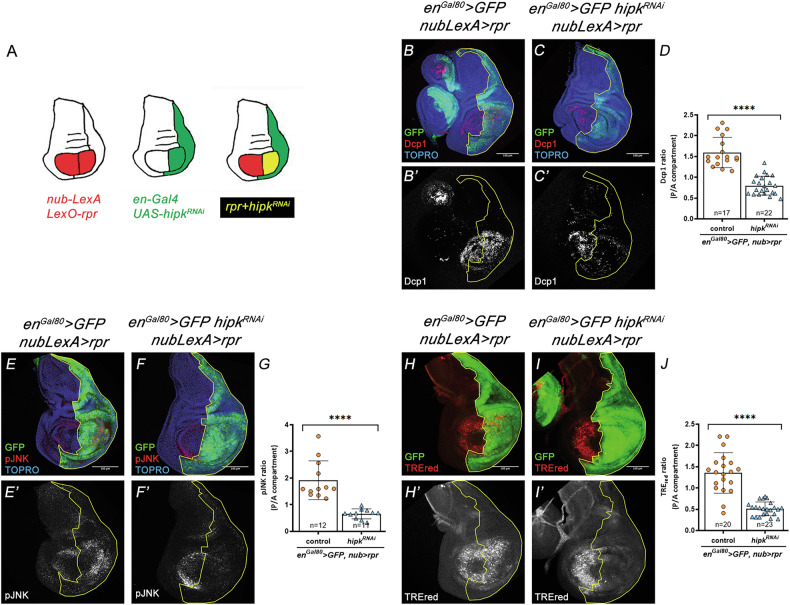
The pro-apoptotic gene *rpr* requires the contribution of *hipk* for full apoptotic response and stimulus of JNK activity. Genotypes on top of the panels. **A** Drawings illustrating the experiments. We have made use of the binary systems Gal4/UAS and LexA/LexO. The *en*-Gal4 driver directs GFP expression and reduces *hipk* levels only in the Posterior (P) compartment, thus discriminating between the Anterior (A) and the P compartments. The *nub*-LexA driver directs *rpr* expression (in red) in the wing pouch, which contains anterior and posterior regions. The combination of the two drivers permits to differentiate regions that contain only *rpr* expression (red), only *hipk* absence (green) or both (yellow). **B**, **B**’, **D** In *en*^*Gal80*^ > *GFP*; *nub-LexA>rpr* discs (the combined expression of *en*-Gal4 and *tub*-Gal80^ts^ is represented, for simplicity, as *en*^*Gal80*^) the entire P compartment is labelled with GFP, and *rpr* is expressed in the Nubbin domain. Staining with the marker Dcp1 (red) shows high apoptotic levels in the entire Nubbin domain, A and P compartments. **C**, **C**’, **D** In contrast, in *en*^*Gal80*^ > *GFP hipk*^*RNAi*^ *nub-LexA>rpr* discs there is a marked reduction of Dcp1 in the posterior Nubbin region. The images in (**E**–**F**’, **G**) and (**H**–**I**’, **J**) illustrate similar experiments demonstrating the effect of the loss of *hipk* function on JNK activity, monitored by the expression of the phosphorylated form of Jun (**E**–**G**) or by the TRE-red marker (**H**–**J**). The scale bar is 100 μm. Data are shown as the means ± SD, the significant level was identified by *p < 0.05; **p < 0.01; ***p < 0.001 and ****p < 0.0001.; ns no significant.

Next, we examined *hipk* role after induction of cell death by the pro-apoptotic gene *reaper* (*rpr*) [9, 11]. Rpr binds to the BIR domain of Diap1 facilitating its proteasomal degradation [39]. This molecular interaction between Rpr and Diap1 secondarily licenses for the activation of initiator and effector caspases, Dronc [40] and Drice [41], therefore inducing cell death [42] (Supplementary Fig. 1). To analyse the response to *rpr* induction of cells in which *hipk* function is compromised, we combined the transcriptional bipartite gene expression systems Gal4/UAS and LexA/LexO [43, 44] (see Material and Methods and drawings in Fig. 2A).

In control wing discs, forced expression of *rpr* in nubbin-expressing cells (in the wing pouch) induced strong cleaved Dcp1 immunoreactivity and JNK activation—both established markers of apoptosis (Fig. 2B, B’, E–E’, H–H’). However, a reduction of *hipk* expression yielded a potent rescue of these features (Fig. 2C–C’, D, F–F’, G, I–I’, J). These results suggested that *hipk* is key for both *rpr*-induced JNK signalling and apoptosis.

The requirement of *hipk* function in the maintenance of JNK activity was further investigated in an experiment in which apoptosis was induced by IR but the execution of the apoptosis program was prevented by overexpressing the effector caspase inhibitor *p35*. In this experimental setting, previous work [18] demonstrated that *dronc*-dependent activation of JNK and persistent proliferative signalling emanating from these cells induces wing imaginal discs overgrowth after irradiation. In line with these observations, irradiated discs expressing *p35* in wing disc posterior cells (P compartment) showed a significant increase in size and ectopic JNK activity, as indicated by the Mmp1 marker [45], compared to non-irradiated control discs (Supplementary Fig. 2A, A’, B, B’, D, E). However, there was no overgrowth and limited JNK activation upon irradiation of P35-expressing cells when *hipk* function is compromised (Supplementary Fig. 2C, C’, D, E).

### *hipk* does not primarily exert its effect through *Diap1*

**Fig. 3:**
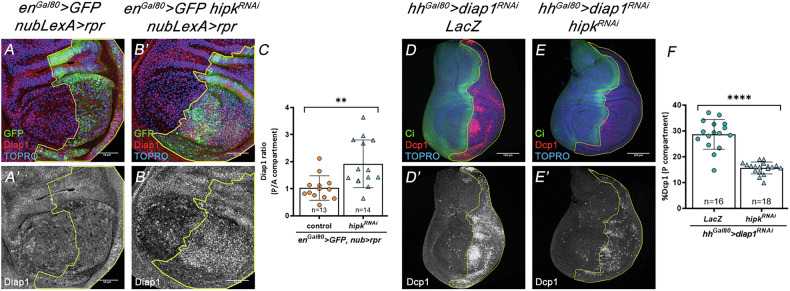
Functional interactions between *hipk* and *diap1.* Genotypes on top of the panels. **A**–**B**’, **C** Reduction of *hipk* function in the posterior region of the Nubbin domain of discs of genotype *en*^*Gal80*^ > *GFP hipk*^*RNA*^ *nub-LexA>rpr* (**B**, **B**’) causes an increase in the amount of Diap1 protein in the P compartment, something not observed in control discs (*en*^*Gal80*^ > *GFP nub-LexA>rpr*) (**A**, **A**’), as indicated by the levels of anti-Diap1 antibody. Quantification in (**C**). **D**, **D**’ Suppression of *diap1* in the P compartment of *hh*^*Gal80*^ >*diap1*^*RNAi*^ *lacZ* discs (the combined expression of *hh*-Gal4 and *tub*-Gal80^ts^ is represented, for simplicity, as *hh*^*Gal80*^) causes a strong apoptotic response, as indicated by the accumulation of the Dcp1 caspase (red). The A/P boundary is delineated by the expression of Ci (green), an A compartment marker. **E**, **E**’ Compromising *hipk* function by RNA interference in the P compartment of *hh*^*Gal80*^ >*diap1*^*RNAi*^ *hipk*^*RNAi*^ larvae results in a significant decrease of Dcp1 levels. Quantification in (**F**). The scale bar is 50 μm in (**A**, **B**’) and 100 μm in (**D**, **E**’). Data are shown as the means ± SD, the significant level was identified by *p < 0.05; **p < 0.01; ***p < 0.001 and ****p < 0.0001; ns no significant.

In addition to its effect on apoptosis and JNK activity, we found that the suppression of *hipk* function in *rp*r-expressing cells caused accumulation of the Diap1 protein (Fig. 3A, A’, B, B’, C), suggesting a role of *hipk* in the *rpr*-mediated degradation of Diap1 [42]. This result also suggested that the diminution of the apoptosis levels observed in the absence of *hipk* function could be due to the maintenance of high levels of Diap1; the Diap1 protein has a key role in preventing the cleavage and subsequent activation of caspases [46], thereby the lack of Diap1 results in massive apoptosis [39, 42, 47–49]. To test this, we first compromised *diap1* expression in the P compartment with an effective RNAi construct [50]. In control discs, in which we reduced *diap1* levels, we detected consistent elevation of Dcp1 (Fig. 3D, D’, F). In contrast, a significant downregulation of Dcp1 was observed by concomitantly reducing *diap1* and *hipk* (Fig. 3E, E’, F). This important result rules out the hypothesis that *hipk* downregulates apoptosis by increasing Diap1 levels, and indicates that Hipk acts downstream *diap1*, possibly facilitating the activation of the caspase pathway.

### *hipk* promotes apoptosis mainly by stabilizing the active form of Dronc

**Fig. 4:**
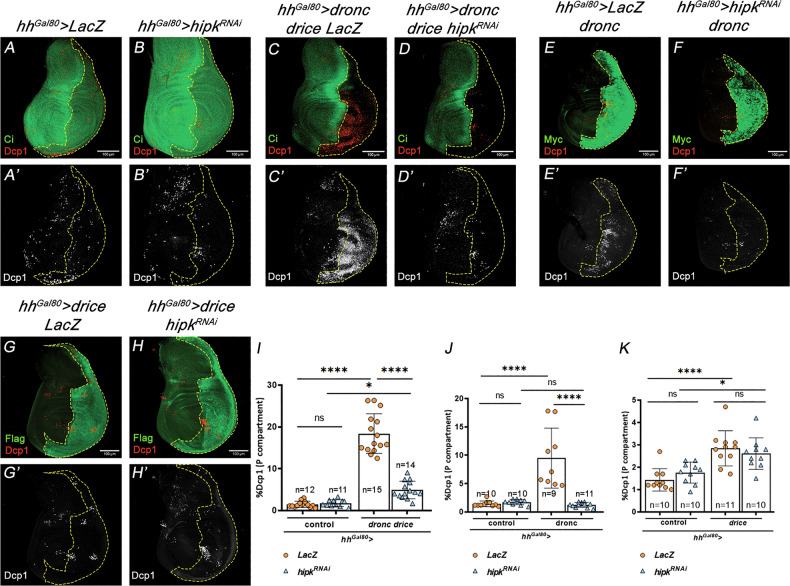
Effect of *hipk* down-regulation on apoptotic levels induced by Dronc and Drice overexpression. Genotypes on top of the panels. **A**–**B**’ The amount of apoptosis (Dcp1, red) in control *hh>lacZ* (A, A’) and *hh>hipk*^*RNAi*^ (**B**, **B**’) discs is similarly low. The Ci antibody marks the anterior compartment. Quantification in (**I**). **C**–**D**’ The joint overexpression of *dronc* and *drice* in the P compartment results in high levels of apoptosis (**C**, **C**’), as indicated by Dcp1 staining, but these are drastically reduced by compromising *hipk* function (**D**, **D**’). The Ci antibody marks the anterior compartment. Quantification in (**I**). **E**–**F**’ Overexpression of *dronc* in the P compartment (marked by an antibody against the Myc tag) shows a moderate increase of apoptosis (**E**, **E**’), which is suppressed by compromising *hipk* function (**F**, **F**’). Quantification in (**J**). **G**–**H**’ Overexpression of *drice* (marked by an antibody against the Flag tag) causes a slight increase of apoptosis (**G**, **G**’), which is not affected by reducing *hipk* activity (**H**, **H**’). Quantification in (**K**). The scale bar is 100 μm. Data are shown as the means ± SD, the significant level was identified by *p < 0.05; **p < 0.01; ***p < 0.001 and ****p < 0.0001; ns no significant.

To explore the hypothesis of a functional interaction between Hipk and the caspase cascade, we analysed the effect of the lack of *hipk* in caspase-activating cells. As expected, our initial control experiments showed that a reduction in the levels of *hipk* did not increase the extremely low amount of physiological apoptosis in the wing disc; a possible reduction of apoptosis cannot be quantified given the extremely low cell death endogenous levels (Fig. 4A–B’, I). We then conducted three experiments that overexpressed *dronc*, *drice*, or both. To overexpress *dronc* and *drice* together we capitalized on a UAS construct in which *dronc* and *drice* cDNAs were concomitantly overexpressed (see Methods). This combined overexpression induced prominent apoptosis in the P compartment of wing discs (Fig. 4C, C, I’). However, such apoptotic response was drastically rescued by downregulating *hipk* (Fig. 4D, D’, I). The single overexpression of either *dronc* or *drice* also induced apoptosis, though to a lesser scale (Fig. 4E, E’, G, G’, J, K). Interestingly, in these experiments Dronc-induced apoptosis, but not Drice-induced apoptosis, was rescued by limiting *hipk* expression (Fig. 4F, F’, J, H, H’, K). Altogether, these data indicated that *hipk* sustains the apoptotic response by likely acting at the level of Dronc.

**Fig. 5:**
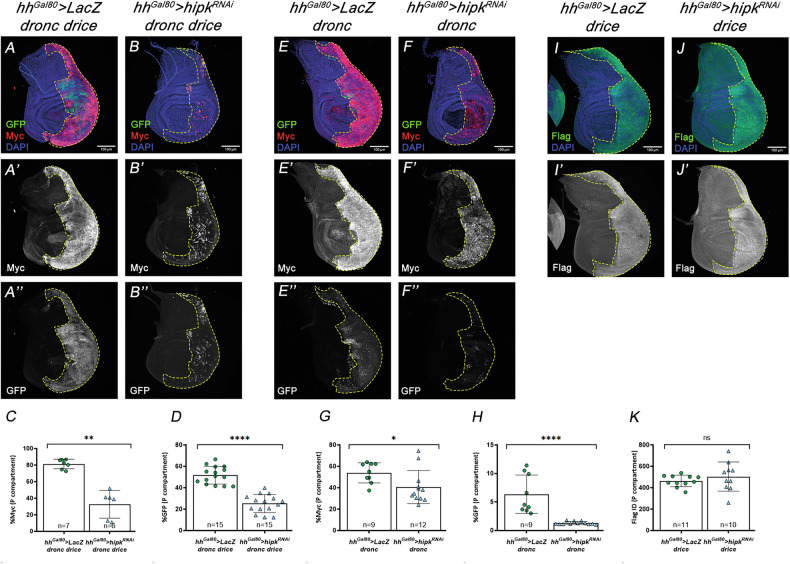
Role of Hipk in the stabilization of the Dronc protein. Genotypes on top of the panels. **A**–**B**” Joint overexpression of *dronc* and *drice* causes an accumulation of total Dronc protein (**A**, **A**’) in the P compartment, as indicated by anti-Myc, as well as of active Dronc protein (**A**, **A**”), as shown by GFP staining, but the lack of *hipk* function substantially reduces both (**B**–**B**”). Quantifications in (**C**, **D**). **E**–**F**” After overexpression of *dronc* in the P compartment, there is also an accumulation of both types of proteins (**E**–**E**”), whose levels are reduced (more clearly for the active GFP one) when *hipk* activity is reduced (**F**–**F**”). Quantifications in (**G**, **H**). **I**–**J**” Overexpression of *drice* in the P compartment results in high levels of Drice protein (labelled with Flag) (**I**, **I**’), which are not altered by compromising *hipk* activity (**J**, **J**’). Quantification in (**K**). The scale bar is 100 μm. Data are shown as the means ± SD, the significant level was identified by *p < 0.05; **p < 0.01; ***p < 0.001 and ****p < 0.0001; ns no significant.

Our previous experiments took advantage of newly generated transgenic lines expressing tagged versions of Dronc and Drice (see Methods) suitable to assess their protein levels and stability upon modulating Hipk expression levels. Specifically, Dronc was fused in-frame with a Myc epitope tag and a modified GFP variant designed to emit fluorescence only upon Dronc-mediated cleavage at an engineered TETDG recognition site introduced within the GFP open reading frame (see Methods and references therein). This construct enables the simultaneous assessment of Dronc protein abundance and activation status by comparing GFP fluorescence with Myc immunostaining. Drice, in turn, was tagged at the C-terminus with a Flag epitope. These constructs allow accurate evaluation of protein levels of the corresponding caspases under overexpression conditions. Remarkably, posterior cells overexpressing Dronc and Drice exhibited strong Myc immunolabelling and GFP fluorescence, indicative of robust Dronc activation (Fig. 5A–A”, C, D). In contrast, reducing Hipk levels led to a marked decrease in both Dronc protein abundance and activation (Fig. 5B–B”, C, D). A milder reduction was also observed in cells overexpressing Dronc under partial Hipk depletion (Fig. 5E–F”, G, H), whereas Drice levels in Drice-overexpressing cells remained unaffected (Fig. 5I–J’, K).

Together, these results strongly support the notion that Hipk enhances the stability of Dronc in its active conformation. Reinforcing this conclusion, lack of *hipk* activity effectively rescues the tissue overgrowth caused by *p35*-expressing cells upon irradiation, which, despite lacking effector caspase activity, still activate Dronc (Supplementary Fig. 2B–D).

**Fig. 6:**
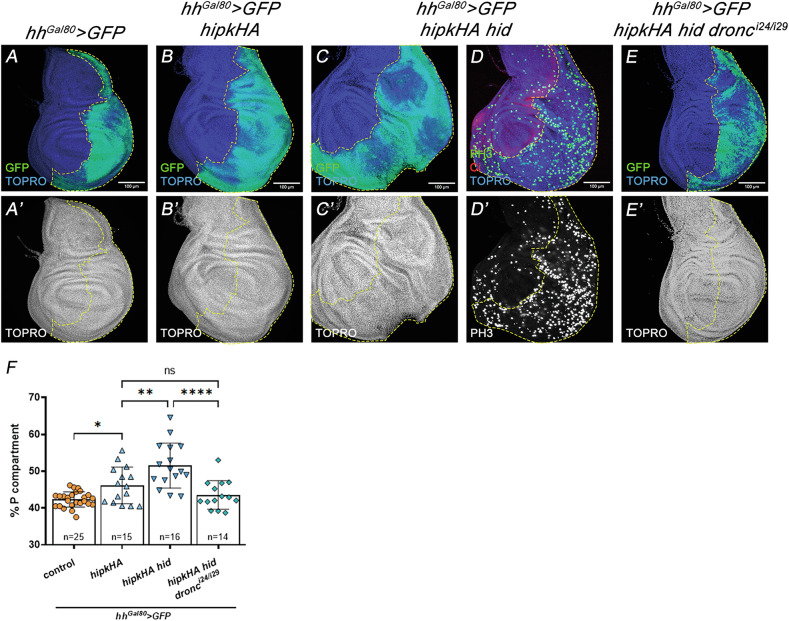
The overgrowth caused by *hipk* is dependent on *dronc* function. Genotypes on top of the panels. **A**, **A**’ Control *hh*^*Gal80*^ > *GFP* wing disc in which the P compartment is labelled with GFP. **B**, **B**’ Overexpression of *hipk*, using a UAS-*hipk-HA* construct expressed in the P compartment, causes a modest overgrowth of the compartment. **C**–**D**’ The concomitant expression of *hid* and *hipk-HA* produces a larger increase in size of the compartment through an increase in cell proliferation, as indicated by higher ph3 staining (in green in (**D**)) in the P compartment. Ci, in red in (**D**), marks the anterior compartment. **E**, **E**’ When both *hid* and *hipk-HA* are expressed but in a *dronc* mutant background (*dronc*^*i24*^/*dronc*^*i29*^), the P compartment size is drastically reduced. Quantifications in (**F**). The scale bar is 100 μm. Data are shown as the means ± SD, the significant level was identified by *p < 0.05; **p < 0.01; ***p < 0.001 and ****p < 0.0001; ns no significant.

Further support for the interaction between *hipk* and *Dronc* comes from experiments of *hipk* overexpression. As previously reported [24, 51, 52], the excess of Hipk caused mild tissue overgrowths (Fig. 6A–B’, F), that were enhanced by the concomitant overexpression of the pro-apoptotic gene *hid* (Fig. 6C–D’, F). However, this phenotype was critically linked to Dronc activity, as it was rescued in a mutant background null for *dronc* (Fig. 6E, E’, F). Similarly, forced expression of *hipk* is sufficient to activate Dronc and cleaved Dcp-1 immunoreactivity, but the absence of *dronc* drastically reduces Dcp1 levels (Supplementary Fig. 3A–E).

### Active Dronc also promotes Hipk protein stability

**Fig. 7:**
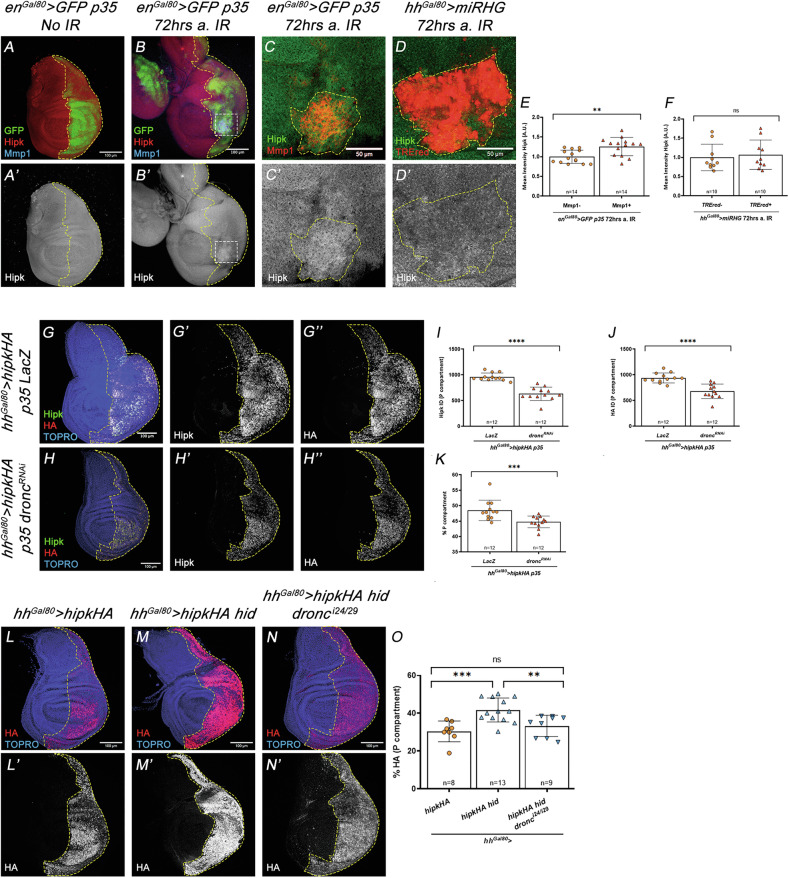
Regulation of Hipk function and levels by pro-apoptotic genes and by *dronc.* Genotypes on top of the panels. **A**, **A**’ In non-irradiated discs, Hipk antibody expression is uniform in the wing disc (red); GFP expression (green) labels the posterior (P) compartment. **B**, **B**’ After (a) IR of *p35*-expressing wing discs, patches of higher Hipk expression are observed (inset). **C**, **C**’ Magnification of the inset shown in (**B**). The delineated area shows JNK activity, as indicated by expression of the Mmp1 marker (red), and increased levels of anti-Hipk signal (**C**’). **D**, **D**’ Portion of the P compartment of an irradiated disc in which activity of pro-apoptotic genes is suppressed by the presence of the *mirRHG* construct [54]. The delineated patch shows JNK activity (TRE-red signal) but Hipk levels (**D**’) are not increased. Quantifications in (**E**, **F**) measure Hipk signal in areas with or without Mmp1 expression (**E**) or with or without TRE-red signal (**F**). **G**–**G**” The overexpression of the *hipk* gene (*hipk-HA* construct) in the P compartment gives rise to an accumulation of the Hipk protein, as measured with the anti-Hipk and anti-HA antibodies. **H**–**H**” If *dronc* expression is reduced in this genetic background, the amount of both anti-Hipk and anti-HA signals, as well as the size of the compartment, are clearly reduced. Quantifications in (**I**–**K**). **L**, **L**’ Forced expression of *hipk-HA* in the P compartment, showing anti-HA signal. **M**, **M**’ The joint overexpression of *hipk-HA* and *hid* strongly increases anti-HA levels in this compartment, but the amount of this signal is drastically reduced in a *dronc* mutant background (**N**, **N**’). Quantifications in (**O**). The scale bar is 50 μm in (**C**–**D**’) and 100 μm in the rest of the panels. Data are shown as the means ± SD, the significant level was identified by *p < 0.05; **p < 0.01; ***p < 0.001 and ****p < 0.0001; ns no significant.

The observations above strongly suggest a determinant role of *hipk* to ensure the correct levels of apoptosis in either developmentally regulated or induced apoptosis. More specifically, our observations suggested that the Hipk protein preferentially affects the stability of Dronc in its active form. Since previous studies have shown that caspases can enhance Hipk2 activity in mammals [53], we sought to investigate whether Hipk might, in turn, be regulated by Dronc. To this end, we first evaluated the Hipk levels in cells expressing *p35*, which cannot complete apoptosis but still activate Dronc after irradiation. Interestingly, in this experimental setting we found groups of *p35*-expressing cells showing significantly elevated levels of Hipk. A closer examination revealed that these cells also activated JNK signalling, as indicated by the Mmp1 upregulation (Fig. 7A–C’, E). This result suggested that ionizing radiation could raise the amount of Hipk in Dronc-activating cells that fail to die. Notably, such upregulation of Hipk did not occur in irradiated discs in which the expression of pro-apoptotic genes was targeted by overexpressing a micro RNA against the proapoptotic genes Rpr, Hid and Grim (mirRHG) [54] (Fig. 7D, D’, F); this occurs despite the fact that the JNK pathway was still upregulated by apoptosis-independent JNK activation [55] (Fig. 7D, D’, F).

These results argue for a role of the apoptotic program in elevating Hipk levels, but do not discriminate if this is a transcriptional or post-transcriptional effect, and do not single out Dronc as the key protein in this regulation. To solve these issues, we forced expression of a *hipk-HA* construct and quantified total Hipk and HA levels in *p35*-expressing cells with either normal or reduced Dronc expression. Intriguingly, absolute levels of Hipk-HA, detected using both anti-HA and a Hipk-specific antiserum were significantly reduced in Dronc-deficient cells with respect to controls (Fig. 7G–J). Consistently, these findings were correlated with a limited ability of Hipk overexpression to induce tissue overgrowth in cells without Dronc (Figs. 6C–F and 7K). To address whether Hipk upregulation was a consequence of impeding the completion of apoptosis via P35 or an effect connected to Dronc, we expressed the UAS-*hipk-HA* construct (along with *hid* but without *p35*) in either wild type or Dronc-deficient cells. The experiment revealed a significant upregulation of HA levels upon *hid* and *hipk-HA* co-expression and a strong reduction of HA levels when *dronc* was absent (Fig. 7L–O). Collectively, these findings support a reciprocal regulatory relationship: Hipk enhances the stability of Dronc, while active Dronc promotes the accumulation of Hipk.

## Discussion

**Fig. 8:**
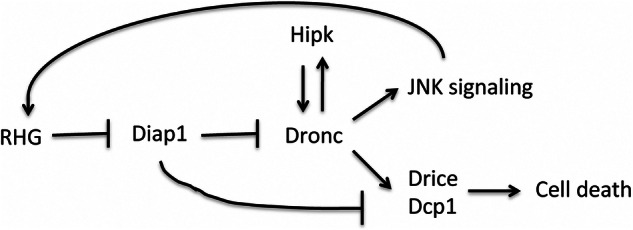
Scheme showing the interaction between Hipk and Dronc in the apoptotic pathway.

In this report we provide compelling evidence that Hipk plays a key role during the execution of apoptosis by stabilizing the active form of Dronc. Given the limited understanding of caspase regulation upon activation, our findings open a new avenue of research with significant implications for caspase biology. Conversely, we also show that Dronc promotes an increase in Hipk expression levels, further amplifying the apoptotic cascade and JNK activation (Fig. 8). This intriguing observation likely reflects a positive feedback loop previously described between Dronc and the JNK pathway [12].

### Hipk is a pro-apoptotic factor that stabilizes active Dronc

Hipk proteins are evolutionarily conserved serine/threonine kinases traditionally associated with fine-tuning transcriptional responses that affect various biological functions, such as cell proliferation, cell fate decisions, and apoptosis [19, 21]. Our results strongly suggest that *Drosophila* Hipk acts as a proapoptotic factor, as its reduced expression significantly diminishes the amount of apoptosis in either developmentally regulated or experimentally induced apoptotic contexts. The implication of *hipk* in developmentally regulated apoptosis has been reported previously [27] and we have confirmed this requirement in the left-right fusion of the abdominal hemisegments and the rotation of the male genitalia. In addition, we show that the activity of JNK is also necessary in both processes, thus pointing to a relevant role of JNK in developmental regulated apoptosis.

We have also demonstrated the involvement of *hipk* in the response to various pro-apoptotic stimuli. Our epistasis experiments show that the loss of Hipk function robustly suppresses apoptosis triggered by either the overexpression of pro-apoptotic factors, the loss of cell death inhibitors such as Diap-1, the combined overexpression of initiator and effector caspases, or by the initiator caspase Dronc alone. In contrast, cell death driven solely by effector caspase overexpression (e.g., Drice) remains largely unaffected by Hipk deficiency. All these experiments position Hipk activity at the level of the initiator caspase Dronc. In parallel, we found in our experiments that Hipk deficiency also compromises JNK activation in apoptosis-induced scenarios, thereby suggesting a Hipk-mediated control of the two Dronc activities: the induction of apoptosis through Dcp1 and Drice, and the amplification of apoptosis and JNK activity through the apoptotic loop [12].

In mammalian systems, Hipk2 also mediates apoptosis, but by direct phosphorylation of P53 [56–58] and/or by facilitating the degradation of its inhibitor, MDM2 [59]. In parallel, members of the Hipk family have been shown to potentiate JNK pathway activation [56] and apoptosis [60, 61] by antagonizing transcriptional repressors of the CtBP family. Thus, in Drosophila and mammals Hipk members regulate apoptosis and JNK signalling, although the molecules involved in such regulation, yet to be unravelled in Drosophila, might be distinct.

Our experiments indicate that Hipk plays a critical role in stabilizing Dronc, most notably the active form of Dronc. In cells exposed to apoptotic stimuli Hipk is substantially required to sustain Dronc stability. The finding that loss of *hipk* causes a diminution of Dronc product may suggest that a primary cause of *hipk* phenotypes is precisely a reduction in the amount of active Dronc protein available to fulfill those roles, what results in partial suppression of Dronc function. Importantly, this regulatory mechanism may differ from those previously described. Thus, protein–protein interactions with Dark [62], and Tango7 [63] have been shown to promote the assembly of protein complexes that enable efficient Dronc activation, while interaction with MyoID localizes Dronc to specific subcellular compartments [64]. Moreover, Hipk probably does not exert its pro-apoptotic function through its canonical role in modulating transcriptional regulation, since many of our experiments were conducted forcing artificially the transcription of Dronc using the Gal4/UAS system. Furthermore, our data raise the possibility that Hipk modulates the stability of active Dronc through phosphorylation—either directly or by influencing upstream regulators involved in its turnover. Such post-translational regulation would not be unexpected, as phosphorylation-based control of caspases has been reported in mammals [65–68], and Dronc in *Drosophila* [69]. To study the biochemical basis of the regulation of Dronc stability by Hipk, however, may be challenging. The difficulty arises from the very low physiological expression levels of Dronc, the fact that Hipk shows a preference to act on the active, and therefore less stable, form of Dronc, and the transient nature of interactions between kinases and their substrates. In summary, although our experiments do not clarify whether Hipk regulates the stability of active Dronc directly or indirectly, they establish a functional link between Hipk and Dronc to achieve the correct levels of apoptosis in development or after pro-apoptotic stimuli. This reveals a novel regulatory pathway modulating Dronc function and broadens our current understanding of caspase biology.

### Hipk, JNK pathway and Dronc key players forming a positive apoptotic feedback loop

The Hipk protein interacts with different transcription factors and other molecules implicated in distinct biological operations [19]. The levels of *Drosophila* Hipk must be tightly regulated since both overexpression or loss of function of *hipk* can induce apoptosis [24]. We have found that pro-apoptotic stimuli like IR cause an elevation of the amount of the Hipk protein, and this increment requires normal function of the apoptotic cascade. This process would ensure that there is a surplus of active Hipk necessary for activation of Dronc. More specifically, our results show that Dronc is needed to maintain Hipk levels, which suggests a mutual interaction between Dronc and Hipk to reciprocally sustain their stability. Further molecular analyses will be required to confirm this conclusion and to elucidate the mechanisms underlying this interaction.

Interestingly, in mammalian cells it has been reported that stress-induced activation of Caspase-6 leads to the proteolytic processing of Hipk2 [53, 61]. Notably, this cleavage event removes an inhibitory C-terminal domain, generating a hyperactive kinase that further amplifies apoptosis. These and our own findings suggest that caspases could be evolutionarily conserved regulators of Hipk, capable of modulating either its protein abundance or activity. This mutual regulation between caspases and Hipk may be critical for amplifying the apoptotic response in diverse cellular contexts across evolution and could represent a targetable axis for future therapeutic interventions.

This mutual Dronc-Hipk interaction also impinges on activation of JNK signalling, a central, evolutionarily conserved regulator of apoptosis [70]. In *Drosophila*, Hipk acts as a positive regulator of the JNK pathway in wing imaginal discs. Its activity is tightly regulated by SUMOylation, and upon loss of SUMO modification (e.g., through Smt3 knockdown) Hipk accumulates in the cytoplasm, enhancing JNK pathway activation and apoptosis [26]. In vertebrates, Hipk proteins appear to function as key positive regulators of JNK signalling and c-Jun phosphorylation, through both direct and indirect mechanisms that are highly context-dependent [56, 60]. Given Hipk’s known role in modulating diverse cellular processes, it is also tempting to speculate that, in addition to JNK signalling, Hipk may also regulate other non-apoptotic functions of Dronc, but further work is needed to validate this hypothesis.

In summary, we have provided evidence that Hipk and caspases engage in a bidirectional positive regulatory relationship that amplifies apoptotic signalling and JNK activation. This molecular crosstalk provides mechanistic insight into a previously reported positive feedback loop between caspase activity and JNK signalling that reinforces the apoptotic fate in *Drosophila* cells [12]. Taken together, prior studies and our current findings delineate a self-reinforcing molecular circuit involving Hipk, JNK signalling, and caspase activation that ensures robust commitment to apoptosis.

## Materials and methods

### Drosophila strains

All the *Drosophila* strains used in this study were raised and maintained on standard medium at 25 °C (see below for the temperature shift experiments). The following *Drosophila* lines were used:

#### Gal4/UAS and LexA/lexO systems

We have used the Gal4/UAS [43] and lexA/lexO [44] systems to express or inactivate different genes in particular locations, in some cases combining the two systems so that two adjacent cell populations with distinct genotypes could be compared.

#### Gal4 lines

*hh-Gal4* [71], *tub-Gal80*^*ts*^ [72], *en-Gal4* (BDSC#30564), *Abd-B*^*LDN*^ (*Abd-B*-Gal4^LDN^) [73], *Eip71CD*-Gal4 [31].

#### lexO line

*lexO-rpr* [74]

#### UAS lines

UAS*-mirRHG* [54], UAS*-GFP* (BDSC#5130), UAS-*hid* [75], UAS-*HA-Hipk2M* [23], UAS-*HA-Hipk3M* [23], UAS-*hipkRNAi* (VDRC KK107857) [24], UAS-*p35* (BDSC#8651), UAS-*cherry* (BDSC#35787), UAS-*lacZ* (BDSC#8529), UAS-Dronc-modified GFP-Myc, MVz-*Drice-Flag-VN*, UAS-*Dronc-GFP-Myc*/MVz-*Drice-Flag-VN* (see below), UAS-*Diap1RNAi* [76], UAS-*droncRNAi* (VDRC #23035).

#### Mutants

*dronc*^*i24*^ (BDSC#91594), *dronc*^*i29*^ (BDSC#91595).

#### Reporter lines

*TRE-red* (BDSC#59011).

A list of the genotypes in the Figures is included as a Supplementary list.

#### Construction of the *nub-lexA* transgene

To generate the *nub*-LexA driver line for *nubbin*, we first amplified 3.8 kb of nubbin genomic regulatory DNA [77] using Taq high-fidelity polymerase. The primers used to perform the PCR were:

FP:CACCCTTCAACTTGTAACTGCTGGCTGCA

RP:GGGGATTGGTCCGAAAAGAGGATAC

PCR products were initially subcloned into the TOPO-TA vector and then transferred as EcoRI fragments into the pBPLexA::GADfluw plasmid (Addgene Plasmid #26232; ref. 78). Correct insertion and sequence fidelity were confirmed by Sanger sequencing. Transgenic flies carrying the construct were generated via PhiC31-mediated integration at the attP40 landing site located at cytological position 22 F on the second chromosome.

### Temperature shift experiments

We made use of the Gal4/Gal80^ts^ system [72] to control the time of expression of different genetics constructs. After an egg lay of 1 day at 25°C, larvae including the genetic combinations *hh-Gal4, tub-Gal80*^*ts*^ or *en*-Gal4 *tub*-Gal80^ts^ were raised at 17 °C and then transferred to a restrictive temperature of 29°C or 31°C for 2 or 3 days before dissection. The combined expression of a Gal4 line, *hh*-Gal4 or *en*-Gal4, and *tub*-Gal80^ts^ is represented, for simplicity, as *hh*^*Gal80*^ and *en*^*Gal80*^, respectively.

### Generation of MVz-Drice-Flag-VN plasmid

We synthesized a wild-type *Drice* cDNA fused at its C-terminus to a Flag tag and the N-terminal half of a split Venus fluorescent protein, using gene synthesis services provided by Twist Bioscience. The resulting fragment was delivered in a pUC51 plasmid backbone. The *Drice*-Flag-VN construct was then excised from pUC51 as a PmeI–KpnI fragment and subcloned into the corresponding sites of the MVz plasmid [79]. Please refer to the plasmid map (Supplementary Fig. 4) for additional details; the full plasmid sequence is available upon request. Transgenic flies carrying the construct were generated via PhiC31-mediated site-specific integration. The construct was inserted at the attP40 site, located at cytological position 25C6.

### Construction of the UAS-Dronc-GFP-Myc

A wild-type *Dronc* cDNA was synthesized (GeneWizz) and fused in-frame to the Suntag and HA-tag peptides at the C-terminal end. To facilitate downstream cloning, additional restriction sites were introduced at both the 5ʹ and 3ʹ ends of the construct, as well as upstream of the tag peptide. The full-length construct was initially subcloned into the pUC57 vector as a *NotI-KpnI* fragment. Subsequently, the vector was digested with *SmaI* and *NheI*, resulting in the removal of the C-terminal Suntag-HA tagging from the wild-type *Dronc* sequence. A modified version of GFP, containing a Myc tag at its C-terminal end, was generated by PCR using the primers listed below. This GFP variant includes a TETDG caspase cleavage site, which replaces the original effector caspase cleavage site described in the original publication [80], which, upon *Dronc*-dependent cleavage, restores GFP to a conformational state compatible with fluorescence emission. The template for the GFP-Myc sequence was described previously [81]. The GFP-Myc PCR product was subsequently cloned in-frame at the C-terminal end of wild-type *Dronc* as a *SmaI-NheI* fragment.

Primers used for GFP-Myc amplification:

- Forward primer: 5ʹ GCTTTAATAAGAAACTCTACTTCAATcccgggtttttcaacgaagggggcATGATCAAGATCGCCACCAGGAAGTACC 3ʹ
- Reverse primer: 5ʹ GATAAAATGTCCAGTGGCGGCAAGCTAGCttacaggtcctcctcgctgatcagcttctgctcGTTAGGCAGGTTGTCCACCCTCATCAGG 3ʹ

The complete construct was then subcloned as a *NotI-XhoI* fragment into a UAS-attB *w+* vector previously linearized with *NotI-PspXI*. Please refer to the plasmid map in Supplementary Fig. 4 for further details; full sequence of the plasmid can be distributed upon request.

Transgenic *Drosophila melanogaster* carrying the UAS-Dronc-GFP-TETDG-Myc construct were generated via PhiC31-mediated site-specific integration. The construct was inserted into the attP site located at the 22A3 locus (Bloomington Drosophila Stock Center, stock #9752).

### Construction of the UAS-Dronc-GFP-Myc/MVz-Drice-Flag-VN dual plasmid

In parallel with the subcloning of the *Dronc*-GFP-Myc fragment into the standard UAS-attB-white⁺ plasmid, we also inserted this construct into a modified version of UAS-attB-white⁺ in which the loxP site upstream of the UAS repeats had been removed by NheI digestion followed by re-ligation. The *Dronc*-GFP-Myc fragment was then subcloned as a NotI–XhoI fragment into this modified plasmid, which had been linearized with NotI–PspXI. From the resulting intermediate plasmid, an NsiI–Dronc-GFP-Myc–NsiI fragment was excised and subcloned into the MVz-Drice-Flag-VN plasmid using the same restriction sites. The resulting dual-expression plasmid enables simultaneous expression of *Dronc*-GFP-Myc and *Drice*-Flag-VN under UAS control. A plasmid map is shown in Supplementary Fig. 4, and the full sequence is available upon request. Transgenic flies carrying the construct were generated via PhiC31-mediated integration at the attP40 landing site, located at cytological position 25C6.

### Imaginal discs staining

Third instar larvae were dissected in PBS and fixed with 4% paraformaldehyde, 0.1% deoxycholate (DOC) and 0.3% Triton X-100 in PBS for 27 min at room temperature. They were blocked in PBS, 1% BSA, and 0.3% Triton, incubated with the primary antibody overnight at 4 °C, washed in PBS 0.3% Triton X-100 and incubated with the corresponding fluorescent secondary antibodies for at least 2 h at room temperature in the dark. They were then washed and mounted in Vectashield mounting medium (Vector Laboratories).

The following primary antibodies were used: rat anti-Ci (DSHB 2A1) 1:50; mouse anti-Mmp1 (DSHB, a combination, 1:1:1, of 3B8D12, 3A6B4 and 5H7B11) 1:50; rabbit anti-Hipk (a gift from E. Verheyen (25)) 1:100; rabbit anti-Dcp1 (Cell Signalling, antibody #9578) 1:200; rabbit anti-Diap1 (a gift from H. Steller (39)) 1:2000.

Fluorescently labelled secondary antibodies (Molecular Probes Alexa-488, Alexa-555, Alexa-647, ThermoFisher Scientific) were used in a 1:200 dilution. DAPI (MERCK) and TO-PRO3 (Invitrogen) were used in a 1:1000 dilution to label nuclei.

### IR treatments

For irradiation experiments, larvae were raised at 17 °C for 3–4 days and then transferred to 31 °C 1 day before irradiation. Then, irradiated larvae were grown at 31 °C for 3 days before imaginal disc dissection. Larvae were irradiated in an X-ray machine Phillips MG102 at the standard dose of 4000 Rads (R).

### Analysis of adult cuticles

Photographs of adult flies were taken with a Leica MZ12 stereomicroscope and a Leica DFC5000 camera, and images were acquired using Leica LAS software (3.7). The images were edited and assembled using Photoshop. Number of animals analysed for each experiment is indicated in legend to Fig. 1.

### Image acquisition, quantifications and statistical analysis

Stack images were captured with a Leica (Solms, Germany) LSM510, LSM710, DB550 B vertical confocal microscope and a Nikon A1R. Multiple focal planes were obtained for each imaginal disc. Quantifications and image processing were performed using the Fiji/ImageJ (https://fiji.sc) and Adobe Photoshop software.

To measure the percentage of positive areas of different markers (%Dcp1, %TREred, %pJNK, %GFP, %Myc), the corresponding positive area was obtained using the “Threshold” tool in ImageJ and then normalized by the area of the compartment (labelled with positive GFP or negative Ci staining). Diap1 ratio (P/A compartment) was calculated as the proportion between the percentage of Diap1 positive areas in the posterior compartment and the anterior compartment.

To quantify the percentage of the posterior compartment, a Z-maximal intensity projection was made for each image. Then, the area of the posterior compartment (labelled with positive GFP or HA staining) was measured by using the “Area” tool and normalized dividing by the total disc area (labelled by TOPRO-3 or DAPI staining).

Flag, Hipk and HA integrated density (ID) were calculated by multiplying the mean intensity (obtained using the “Threshold” tool in ImageJ) and the area of the posterior compartment (labelled by positive Flag, Hipk or HA staining. ID data were normalized by the total disc area (labelled by DAPI or TOPRO staining).

Statistical analysis was performed using the GraphPad Prism v8 software (https://www.graphpad.com). When comparing between two groups, a non-parametric Student’s *t* test (Mann–Whitney’s test) was used. To compare between more than two groups, a non-parametric, one-way ANOVA test (Kruskal–Wallis test) was used. Sample size was indicated in each graph. Error bars in the graphs represent the standard deviation (SD). *p*-values obtained in each statistical analysis were represented in the graphs according to the following nomenclature: *p < 0.05; **p < 0.01; ***p < 0.001 and ****p < 0.0001.

## Supplementary information

Supplementary Figures

Supplementary list of genotypes

## Data Availability

All data are available from the corresponding authors upon reasonable request.
