# Supplementary material for: The homeodomain-interacting protein kinase Hipk promotes apoptosis by stabilizing the active form of Dronc
Source: Cell Death Discov. 2025 Dec 16;12:53. doi: 10.1038/s41420-025-02916-9 (PMC12848060; doi:10.1038/s41420-025-02916-9)
Supplement: Supplementary file 1 — Supplementary Figures [file 41420_2025_2916_MOESM1_ESM.docx]

**
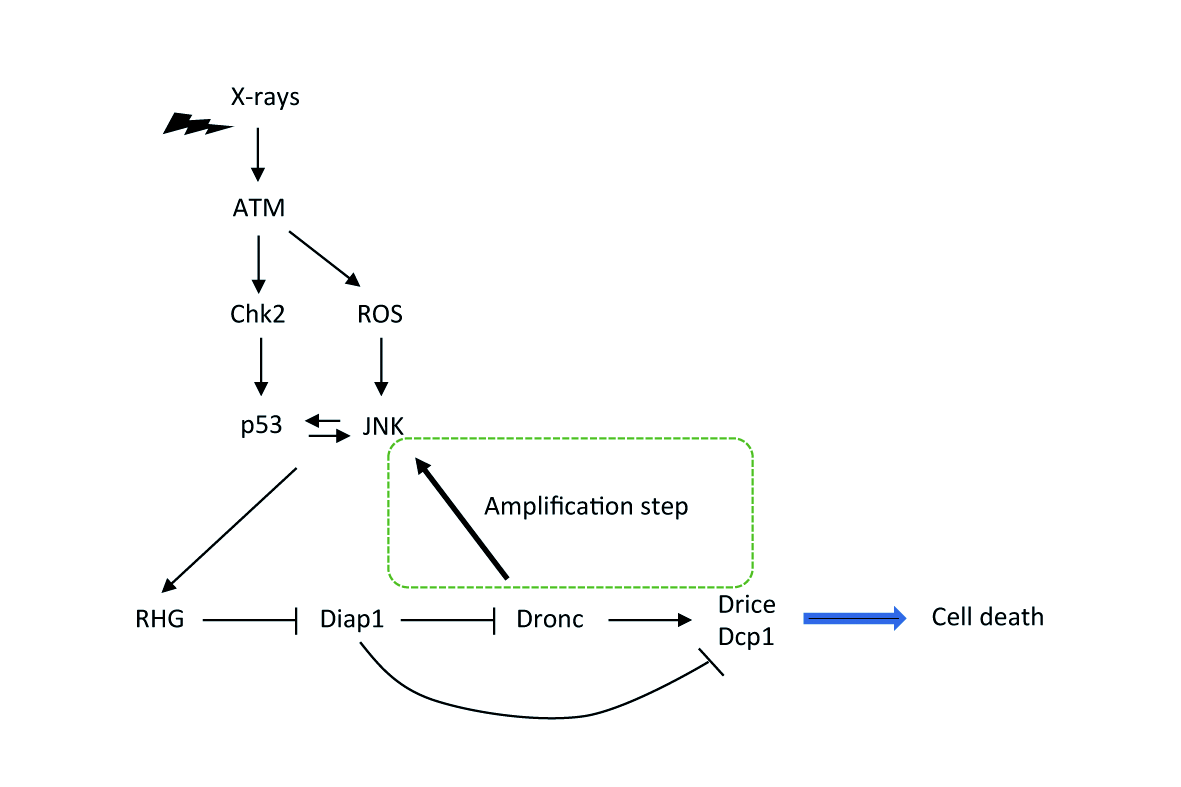
Figure S1**. **Simplified version of the apoptosis program of *Drosophila*, as triggered by X-rays**.

The DNA double-strand breaks caused by the irradiation activates the Ataxia Telengiestasia Mutated (ATM) kinase, which in turn activates the Checkpoint2 (Chk2) kinase and also induces the production of Reactive Oxygen Species (ROS). These events trigger the function of *p53* and of the Jun N-Terminal Kinase (JNK) pathway, known to stimulate each other. The expression of *p53*/JNK transcriptionally activates the pro-apoptotic genes *reaper (rpr), head involution defective (hid)* and *grim*, which cause ubiquitination of the Drosophila inhibitor of apoptosis protein1 (Diap1) and allow activation of the apical caspase Dronc and subsequently of the effector caspases Drice and Dcp1. In addition of activating the effector caspases, Dronc stimulates JNK levels, thus establishing an amplification loop, necessary for the full apoptotic response. The amplification step is squared for emphasis.

**
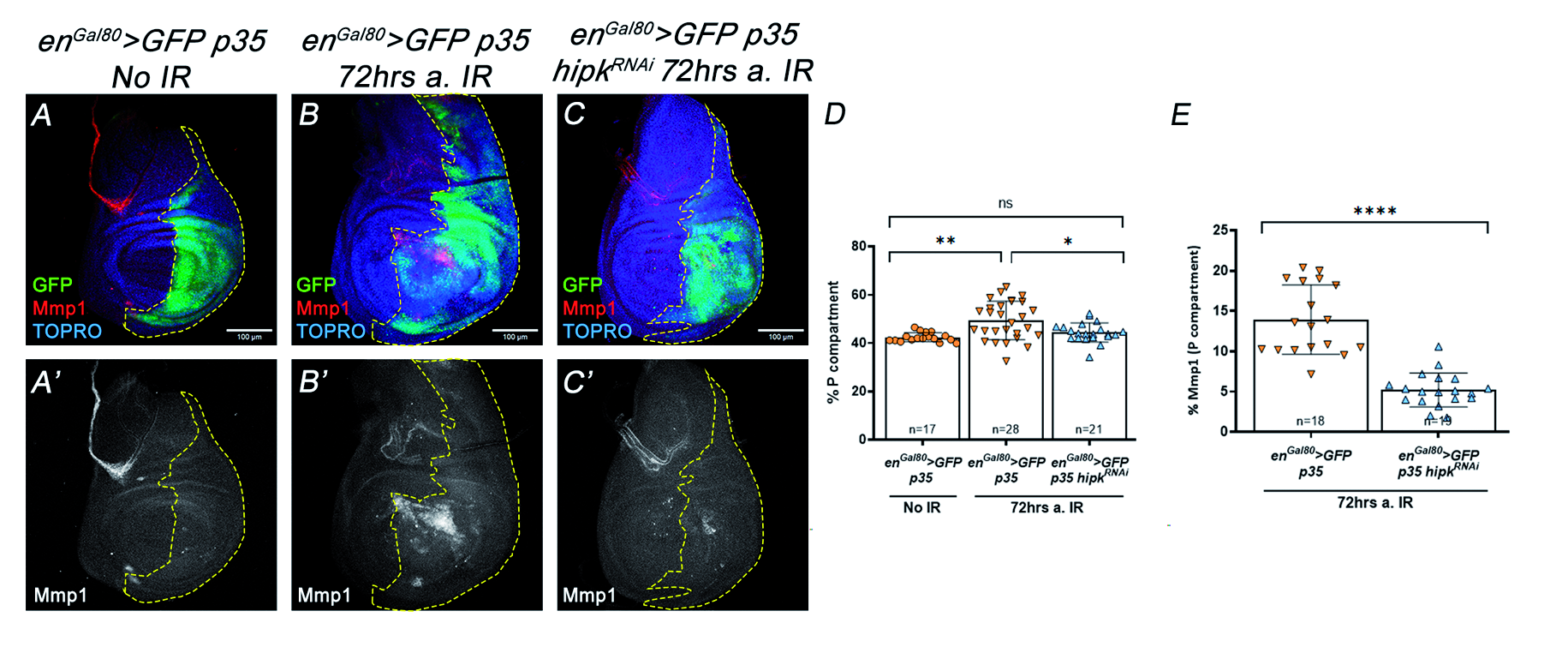
Figure S2. *hipk* is required to maintain JNK function and size increase after irradiation (IR)**

Genotypes on top of the panels. (A, A’) Non-irradiated discs show no JNK activity, monitored here by the presence of Metalloprotease 1, Mmp1 (in red), a target of JNK. (B, B’) In irradiated discs of the *en^Gal80^*>*GFP* *p35* genotype, the presence of the baculovirus protein P35 in the P compartment allows the survival of cells in which JNK has been induced by IR, thus increasing Mmp1 signal. Size of the compartment is also increased. (C, C’) In *p35*-expressing irradiated discs in which *hipk* function is reduced by the expression of a *hipk^RNAi^* construct, JNK activity and compartment size are much diminished. Quantifications in D, E. The scale bar is 100 μm. Data are shown as the means ± SD, the significant level was identified by **p*<0,05; ***p*<0,01; ****p*<0,001 and *****p*<0,001.; ns: no significant.


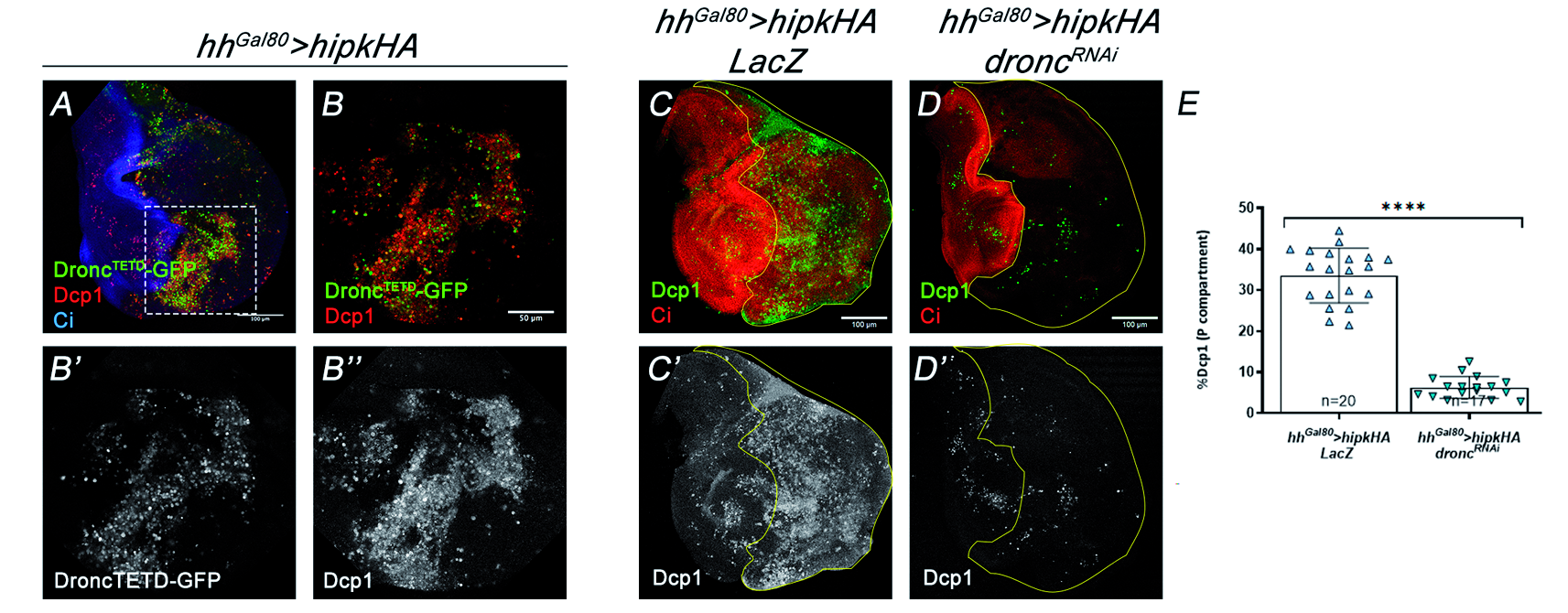


**Figure S3. Overexpression of *hipk* results in *dronc*-dependent apoptosis**

Genotypes on top of the panel. (A-B’’) Overexpression of *hipk-HA* induces the expression of the Dronc-activity reporter *dronc^TETD^-GFP* and Dcp1 antibody signal (A). B-B’’ are amplifications of the inset in A. (C-D’’) The Dcp1 signal induced by overexpression of *hipk-HA* (C, C’) is significantly reduced by the simultaneous inactivation of *dronc* (D, D’). Ci, in red, marks the A compartment (in blue in A, in red in C, D). Quantifications in E. The scale bar is 50μm in B-B’’ and 100 μm in A, C-D’. Data are shown as the means ± SD, the significant level was identified by **p*<0,05; ***p*<0,01; ****p*<0,001 and *****p*<0,001.; ns: no significant.

**
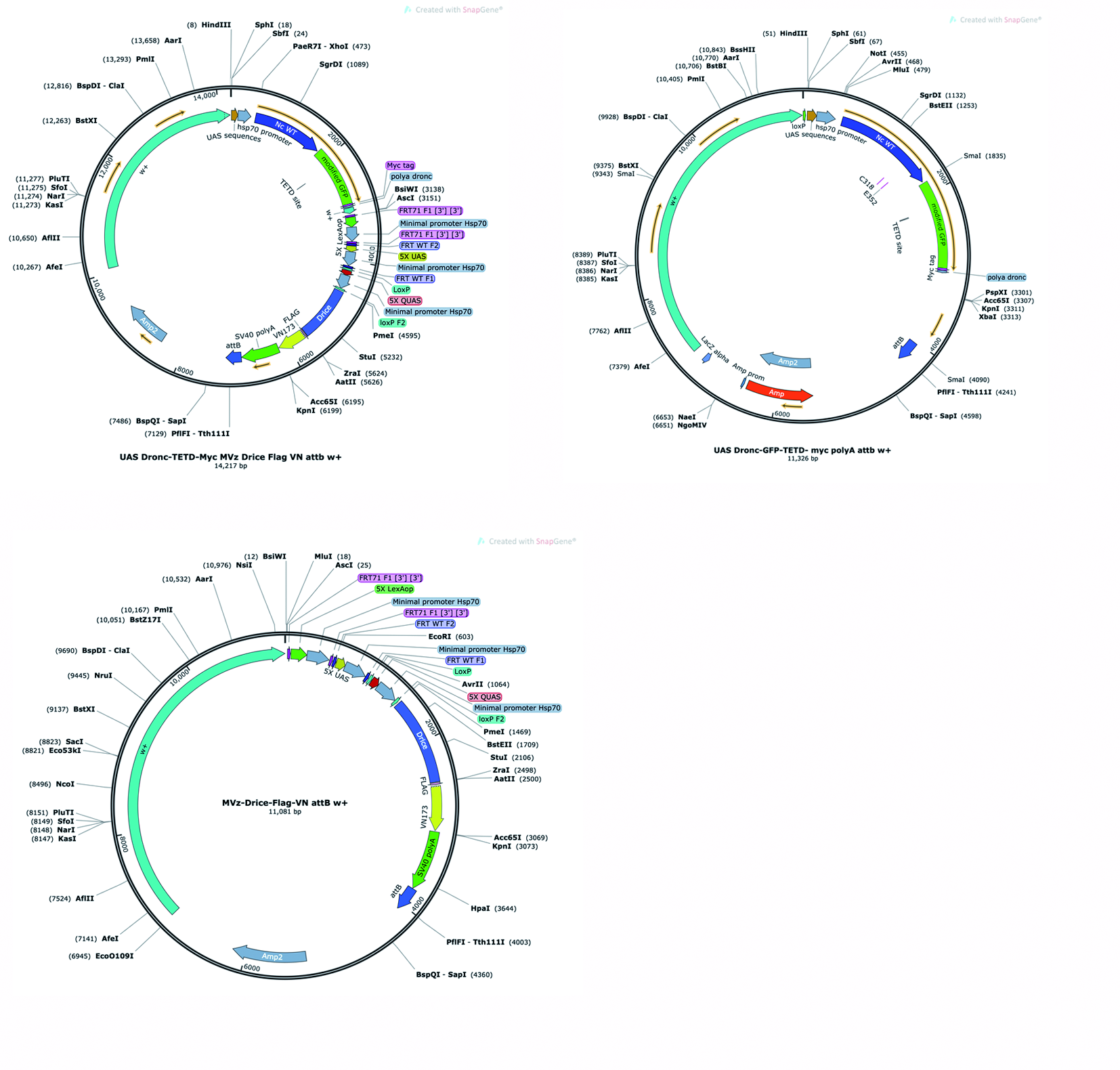
Figure S4. Plasmid maps of the MVz-Drice-Flag-VN, UAS-Dronc-GFP-Myc and UAS-Dronc-GFP-Myc/MVz-Drice-Flag-VN plasmids.**

a) MVz-Drice-Flag-VN: This plasmid was used to generate transgenic flies capable of overexpressing a tagged form of Drice containing a Flag tag and the N-terminal fragment of Venus GFP. Overexpression can be driven by any of the available Drosophila binary systems, including Gal4/UAS, LexA/LexAop, and QF/QUAST. The plasmid backbone was described in  Ref. 79.

b) UAS-Dronc-GFP-TETDG-myc: This plasmid was used to generate flies that overexpress a tagged form of Dronc containing a myc tag and a modified GFP. The GFP variant fluoresces only after cleavage by Dronc at the engineered TETDG site, which replaces the original effector caspase cleavage site described in the original publication PMID: 28870988. Comparing the GFP signal with the myc immunostaining allows for the evaluation of both the absolute protein levels of Dronc and its activation status.

c) UAS-Dronc-GFP-TETDG-myc / MVz-Drice-Flag-VN: This fusion plasmid was used to generate flies capable of simultaneously overexpressing the constructs described above. Note that while Dronc expression is restricted to the Gal4/UAS system, the tagged Drice can be overexpressed using any of the available binary transcriptional systems.
