# Supplementary material for: The homeodomain-interacting protein kinase Hipk promotes apoptosis by stabilizing the active form of Dronc
Source: Cell Death Discov. 2025 Dec 16;12:53. doi: 10.1038/s41420-025-02916-9 (PMC12848060; doi:10.1038/s41420-025-02916-9)
Supplement: Supplementary file 2 — Supplementary list of genotypes [file 41420_2025_2916_MOESM2_ESM.docx]

**Figure 1** A) Eip71CD-Gal4/+; UAS-cherry/+

B) Eip71CD-Gal4/UAS-P35

C) UAS-bskDN; Eip71CD-Gal4/+

D) Eip71CD-Gal4/UAS-hipkRNAi

A) Abd-B^LDN^-Gal4/UAS-cherry

B) UAS-P35/+; Abd-B^LDN^-Gal4/+

C) UAS-bskDN; Abd-B^LDN^-Gal4/+

D) UAS-hipkRNAi/+; Abd-B^LDN^-Gal4/+

**Figure 2**  B, E, H) en-Gal4 UAS-GFP/+; nub-lexA tub-Gal80^ts^/lexO-rpr

C, F, I) en-Gal4 UAS-GFP/UAS-hipkRNAi; nub-lexA tub-Gal80^ts^/lexO-rpr

**Figure 3** A) en-Gal4 UAS-GFP/+; nub-lexA tub-Gal80^ts^/lexO-rpr

B) en-Gal4 UAS-GFP/UAS-hipkRNAi; nub-lexA tub-Gal80^ts^/lexO-rpr

D) UAS-lacZ/UAS-Diap1RNAi; hh-Gal4 tub-Gal80^ts^/+

E) UAS-hipkRNAi/UAS-Diap1RNAi; hh-Gal4 tub-Gal80^ts^/+

**Figure 4** A) UAS-lacZ/+; hh-Gal4 tub-Gal80^ts^/+

B) UAS-hipkRNAi/+; hh-Gal4 tub-Gal80^ts^/+

C) UAS-lacZ/UAS-dronc-drice; hh-Gal4 tub-Gal80^ts^/+

D) UAS-hipkRNAi /UAS-dronc-drice; hh-Gal4 tub-Gal80^ts^/+

E) UAS-lacZ/UAS-dronc; hh-Gal4 tub-Gal80^ts^/+

F) UAS-hipkRNAi /UAS-dronc; hh-Gal4 tub-Gal80^ts^/+

G) UAS-lacZ/UAS-drice; hh-Gal4 tub-Gal80^ts^/+

H) UAS-hipkRNAi /UAS-drice; hh-Gal4 tub-Gal80^ts^/+

**Figure 5**  A) UAS-lacZ/UAS-dronc-drice; hh-Gal4 tub-Gal80^ts^/+

B) UAS-hipkRNAi /UAS-dronc-drice; hh-Gal4 tub-Gal80^ts^/+

E) UAS-lacZ/UAS-dronc; hh-Gal4 tub-Gal80^ts^/+

F) UAS-hipkRNAi /UAS-dronc; hh-Gal4 tub-Gal80^ts^/+

I) UAS-lacZ/UAS-drice; hh-Gal4 tub-Gal80^ts^/+

J) UAS-hipkRNAi /UAS-drice; hh-Gal4 tub-Gal80^ts^/+

**Figure 6** A) UAS-GFP/+; hh-Gal4 tub-Gal80^ts^/+

B) UAS-GFP/+; hh-Gal4 tub-Gal80^ts^/UAS-hipk-HA

C, D) UAS-GFP/UAS-hid tub-Gal80^ts^; hh-Gal4/UAS-hipk-HA

E) UAS-GFP UAS-hipk-HA /UAS-hid tub-Gal80^ts^; hh-Gal4 dronc^i29^/

dronc^i24^

**Figure 7** A, B, C) en-Gal4 UAS-GFP/+; tub-Gal80^ts^/UAS-p35

D) UAS-mirRHG/+; hh-Gal4 tub-Gal80^ts^/+

G) UAS-p35/UAS-lacZ; hh-Gal4 tub-Gal80^ts^/ UAS-hipk-HA

H) UAS-p35/UAS-droncRNAi; hh-Gal4 tub-Gal80^ts^/ UAS-hipk-HA

L) hh-Gal4 tub-Gal80^ts^/ UAS-hipk-HA

M) UAS-hid tub-Gal80^ts^ /+; hh-Gal4/ UAS-hipk-HA

N) UAS-hid tub-Gal80^ts^ / UAS-hipk-HA; hh-Gal4 dronc^i29^/ dronc^i24^

**Figure S2** A, B) en-Gal4 UAS-GFP/+; tub-Gal80^ts^/UAS-p35

C) en-Gal4 UAS-GFP/UAS-hipkRNAi; tub-Gal80^ts^/UAS-p35

**Figure S3** A, B) hh-Gal4 tub-Gal80^ts^/ UAS-hipk-HA

C) UAS-lacZ/+; hh-Gal4 tub-Gal80^ts^/ UAS-hipk-HA

D) UAS-droncRNAi/+; hh-Gal4 tub-Gal80^ts^/ UAS-hipk-HA
